# Kinesiophobia and Clinical Outcomes in People with Chronic Low Back Pain: A Cross-Sectional Study

**DOI:** 10.3390/jcm15103972

**Published:** 2026-05-21

**Authors:** Maram Yahya Asiri, Rania N. Almeheyawi, Doaa S. ALSharif, Fahad H. Alshehri, Jamilah Zabarmawi, Weaam Alghamdi, Ashwag Alwagdani, Hosam Alzahrani

**Affiliations:** 1 Department of Rehabilitation, King Faisal Medical Complex, Ministry of Health, Taif 26521, Saudi Arabia; 2 Department of Physical Therapy, College of Applied Medical Sciences, Taif University, Taif 21944, Saudi Arabia; ralmeheyawi@tu.edu.sa (R.N.A.);; 3 Department of Physical Therapy, King Abdul Aziz Hospital, Ministry of Health, Makkah 24226, Saudi Arabia; jzbrmawy@moh.gov.sa; 4 Department of Rehabilitation, King Fahd General Hospital, Ministry of Health, Jeddah 23344, Saudi Arabia; wralghamdi@moh.gov.sa

**Keywords:** Kinesiophobia, chronic back pain, pain intensity, functional disability, quality of life, fear of movement

## Abstract

**Background/Objective**: Kinesiophobia is a major fear-avoidance concept in chronic low back pain (CLBP); however, its independent contribution to pain, disability, and health-related quality of life (HRQoL) beyond sociodemographic and clinical variables remains unclear. This study aimed to evaluate the associations between kinesiophobia and patient-reported outcomes in adults with chronic low back pain regarding (i) pain intensity, (ii) functional disability, and (iii) HRQoL. **Methods**: This cross-sectional study included 298 participants with CLBP (average age 38.7 ± 13.2 years; 58.0% female). Kinesiophobia was evaluated using the Tampa Scale of Kinesiophobia (range, 17–68). Outcomes were pain intensity (Numerical Pain Rating Scale; 0–10), functional disability (Roland–Morris Disability Questionnaire; 0–24), and HRQoL (RAND-36; 0–100). Two multivariable linear regression models were used per outcome. Model 1 was adjusted for sex and age, and Model 2 was additionally adjusted for BMI, marital status, education, employment, smoking status, and chronic disease. Hierarchical regression analysis evaluated the incremental variance explained by kinesiophobia (ΔR^2^) when entered after all covariates. Effects were reported per 10-point increase in Tampa score, with 95% confidence intervals (CI). **Results**: In the fully adjusted models, higher kinesiophobia was associated with greater pain intensity (B = +1.17 points per 10 Tampa; 95% CI 0.55–1.79, *p* < 0.001), greater disability (B = +3.24 points; 95% CI 2.05–4.43; *p* < 0.001), and lower HRQoL (B = −7.98 points; 95% CI −11.1–−4.81; *p* < 0.001). Hierarchical regression analyses showed that kinesiophobia explained additional variance in pain (ΔR^2^ = 0.11), disability (0.12), and HRQoL (0.11), all *p* < 0.001. **Conclusions**: In adults with CLBP, kinesiophobia was associated with greater pain intensity, functional disability, and lower HRQoL, accounting for 11–12% of variance in each outcome beyond demographic and clinical covariates. These findings support routine assessment of kinesiophobia and justify longitudinal and interventional studies to determine temporal relationships and treatment effects.

## 1. Introduction

Lower back pain (LBP) is a common musculoskeletal disorder worldwide and a major cause of disability [1,2]. Although most episodes of acute LBP resolve spontaneously within weeks, a considerable proportion of individuals fail to recover, and their condition progresses to chronic low back pain (CLBP) [3]. In addition to pain, CLBP often restricts movement, resulting in psychological and social challenges that make life and work difficult. CLBP is more than just pain in the lower back; it affects millions of people and is a huge burden on healthcare systems and economies. Addressing this problem involves moving beyond physical symptoms and exploring the psychological factors that sustain the condition. The coexistence of psychological and physical symptoms highlights the importance of a holistic approach to pain management that goes beyond physical symptoms and targets the psychological factors that sustain the condition [4,5].

Within the biopsychosocial framework, kinesiophobia and fear of movement due to anticipation of injury or reinjury are considered major barriers to recovery. This debilitating fear has been described by patients’ perception that avoiding movement is a rational and appropriate behavior aimed at avoiding further harm or pain [4]. Therefore, fear triggers avoidance behaviors that paradoxically worsen pain, disability, and somatic awareness, among other detrimental effects [6]. Therefore, understanding this association is critical for disrupting the cycle of fear and avoidance, subsequently facilitating functional recovery and ultimately enhancing the overall well-being of individuals with CLBP. Existing evidence supports a conservative, biopsychosocial approach for CLBP. Recent reviews have shown that exercise, physical activity, manual therapy, and pain neuroscience education can improve pain and disability outcomes, especially when used within multimodal care [7,8,9].

The existing literature has demonstrated associations between kinesiophobia and various important patient outcomes in CLBP, such as pain, disability, and health-related quality of life (HRQoL); however, various limitations exist. For example, although Comachio et al. (2018) [10] demonstrated correlations between kinesiophobia and pain intensity, disability, and HRQoL, their analyses relied only on bivariate correlations and did not adjust for potential confounders. Similarly, Antunes et al. (2013) [11] found associations among kinesiophobia, pain intensity, and quality of life; however, they did not assess the independent association of kinesiophobia beyond other psychosocial or demographic factors. Doménech-Fernández et al. (2025) [12] employed multivariate models and found that kinesiophobia, catastrophizing, and sex explained 35% of the variance in disability, but they did not investigate the unique variance attributable to kinesiophobia. Other studies, including those by Torres Cruz et al. (2025) [13], evaluated relationships with specific subpopulations (e.g., older individuals or patients under tertiary care) or focused on predictors of kinesiophobia rather than on outcomes. Additionally, there is limited evidence on the extent of the incremental explanatory value of kinesiophobia in models of pain, disability, and HRQoL beyond demographic and clinical covariates. Together, these limitations highlight the need for methodologically robust, adjusted analyses in varied clinical settings and populations to better understand the independent association of kinesiophobia with CLBP burden.

Therefore, this study aimed to examine the relationship between kinesiophobia and patient-reported outcomes in adults with CLBP, considering (i) pain intensity, (ii) functional disability, and (iii) HRQoL in adults with CLBP. We hypothesized that higher levels of kinesiophobia would be associated with greater pain intensity and disability, and poorer HRQoL.

## 2. Materials and Methods

### 2.1. Design and Setting

This cross-sectional study was conducted in accordance with STROBE guidelines. Ethical approval was granted by the Scientific Research Ethics Committee at Taif University (IRB no. 45-040, approved in October 2023) and the Directorate of Health Affairs, Taif (IRB no. HAP-02-T-067, approved in October 2023). The study was conducted in accordance with the Declaration of Helsinki (1975, revised in 2013). All study participants provided written informed consent prior to data collection. The inclusion criteria were adult individuals aged 18 years or older, diagnosed with CLBP, and with a pain intensity score of at least 3 of 10 as assessed using the Numerical Rating Scale (NRS). This study excluded those with LBP attributable to a specific pathological cause (e.g., spinal surgery, fracture), pregnancy, and cognitive impairments/psychiatric conditions. It also excluded those with any other medical condition that could possibly impact pain perception, functional disability, or HRQoL outcomes, and/or those who are unable to read or understand Arabic. For the purpose of this study, CLBP was defined as pain located in the region between the lower rib margin and the gluteal folds, with or without referred pain, persisting for a minimum of 3 months, and not attributable to a specific pathology.

Data were collected from November 2023 to April 2024. A non-random convenience sampling was employed in recruiting participants. Eligible participants with chronic low back pain were recruited from private clinics and public hospitals in Saudi Arabia. During the data collection period, consecutive potential participants were approached, screened for eligibility and invited to participate. Informed consent was obtained from all those who met the inclusion criteria and agreed to participate prior to enrollment.

### 2.2. Sample Size Calculation

To determine the required sample size, we used GPower (version 3.1) software with multiple linear regression analysis. Assuming a small-to-moderate effect size (Cohen’s f^2^ = 0.05), 80% power, a two-tailed alpha level of 0.05, and allowing for as many as eight covariates, the minimum required sample was 168 participants. However, to ensure that the study had sufficient power and to account for possible missing or incomplete data, we recruited 298 participants.

### 2.3. Study Tools and Instruments

Data were collected by experienced physical therapists working in both public hospitals and private clinics using electronic surveys administered to the patients. The questionnaire was self-administered, and help was offered only in case of any technical problems. In order to avoid missing data, questionnaire completion was checked at the time of data collection, and participants were asked to complete any unanswered items before submission. Consequently, no missing data were present in the final dataset. The survey comprised two parts. The first part of the survey collected participants’ demographic variables such as sex, age, marital status, height, weight, employment status, smoking status, and presence of chronic diseases. The second part collected data on variables related to kinesiophobia, pain intensity, functional disability, and HRQoL.

### 2.4. Kinesiophobia

Kinesiophobia was assessed using the valid and reliable Tampa Scale of Kinesiophobia [14]. It consists of 17 items, and each question has four response options (strongly disagree, disagree, agree, or strongly agree) with scores ranging from 1 to 4 points. The individual scores of Items 4, 8, 12, and 16 were inverted before the total score was calculated. The total score ranged from 17 to 68. Higher scores indicated greater fright of movement with a higher degree of kinesiophobia, which was defined by a cut-off score of 37. The Arabic version of the Tampa Scale of Kinesiophobia (TSK) has been shown to be valid and reliable for assessing the fear of pain in Arabic-speaking populations [15].

### 2.5. Pain Intensity

Pain intensity was assessed using the valid and reliable Numeric Pain Rating Scale (NPRS) [16]. This is an 11-point numeric pain scale ranging from 0 (no pain) to 10 (worst pain ever) at the time of assessment. The Arabic version of the NPRS is a valid and reliable tool for assessing pain intensity in Arabic-speaking populations [17].

### 2.6. Disability

Disability was assessed using the valid and reliable Roland–Morris Disability Questionnaire (RMDQ) [18]. It consists of 24 questions that focus on regular activities of daily living. Each affirmative answer was awarded one point, and the final score was determined as the total number of points. The total scores range from 0 (no disability) to 24 (maximal disability). We used the Arabic version of the RMDQ, which was cross-culturally adapted and psychometrically validated for use in Arabic-speaking populations [19].

### 2.7. Health-Related Quality of Life

Health-related quality of life was assessed using the valid and reliable RAND 36-Item Health Survey [20]. It assesses eight health concepts using multi-item scales (35 items): physical functioning (10 items), role limitations caused by physical health problems (4 items), emotional role limitation (3 items), social functioning (2 items), emotional well-being (5 items), energy/fatigue (4 items), pain (2 items), and general health (5 items). Each domain was scored from 0 to 100, and the total score was computed by averaging the scores of the eight domains, with higher scores indicating better health. The Arabic version used in this study demonstrated good reliability and equivalence to the English version [21].

### 2.8. Statistical Analysis

Statistical analyses were performed using IBM SPSS Statistics 27 software. Descriptive statistics were employed to describe participants characteristics. Continuous variables were reported as mean ± standard deviation (SD), and categorical variables as frequency (percentage). Two ordinary least-squares regression analyses were conducted for each outcome: pain intensity, functional disability, and HRQoL. Model 1 included kinesiophobia, sex and age as basic demographic factors. Model 2 additionally included BMI, marital status, smoking, education, employment, and chronic disease status. The ordinary least-squares regression analyses were used as each outcome was treated as a continuous variable. Covariates were selected a priori, considering their clinical relevance and potential for confounding the association between kinesiophobia and the study outcomes [22,23,24,25]. Regression coefficients were scaled for every 10-point increase in the Tampa Scale score to make it easier to interpret clinically. To establish the unique contribution of kinesiophobia to pain intensity, a hierarchical regression analysis was conducted by entering the covariates first, followed by the Tampa Scale scores. The change in explained variance and its corresponding F-change statistics were used to measure incremental predictive power. All tests were two-tailed with a 5% level of significance. Effect sizes were reported along with 95% confidence intervals. The adjusted R^2^ values explain the overall model fit, whereas R^2^ isolates the variance attributable to kinesiophobia.

## 3. Results

This study included 298 patients with CLBP (Table 1). The average age of the participants was 38.7 ± 13.2 years. Most participants were female (58%), had normal weight (33.2%), were married (55.4%), held a bachelor’s degree (67.4%), were either unemployed or retired (51.3%), were nonsmokers (81.9%), and did not report any chronic disease (71.1%). Participants had an average kinesiophobia score of 44.5 ± 5.4, pain intensity of 6.5 ± 2.1, functional disability of 7.7 ± 5.7, and HRQoL of 47.8 ± 12.9.

Table 2 shows that kinesiophobia was significantly correlated with all three outcomes. In the minimally adjusted model (Model 1), the association between the Tampa Scale and the outcomes is as follows: for every 10-point increase in the Tampa Scale, the pain intensity (10-point NPRS) increases by 1.19 (95% Confidence Interval (CI) 0.61–1.77, *p* < 0.001), functional disability (24-item RDC) increases by 3.27 (95% CI 2.14–4.41, *p* < 0.001), and HRQoL (RAND-36) decreases by 7.99 (95% CI −11.0–−4.98, *p* < 0.001). When the fully adjusted model (Model 2) is used, the estimates of the association between the Tampa Scale and the outcomes remain similar, with the unstandardized coefficients being 1.17, 3.24, and −7.98 for pain intensity, functional disability, and HRQoL, respectively. The overall model fit was comparable across outcomes (adjusted R^2^ ≈ 0.14–0.18).

Hierarchical regression analysis (Table 3) showed that adding kinesiophobia to the maximally adjusted model was associated with increase in the explained variance beyond all covariates. After entering all covariates (Step 1), adding the Tampa Scale (Step 2) increased R^2^ by 0.11 for pain intensity, 0.12 for functional disability, and 0.11 for HRQoL. Each ΔR^2^ was significant (F-change > 36.5, *p* < 0.001), demonstrating that kinesiophobia was associated with additional variance in each outcome, even after controlling for all other covariates.

## 4. Discussion

In the present study on adults with CLBP, the results reported that higher kinesiophobia was associated with poorer patient-reported outcomes. For every 10-point increase on the TSK, the pain intensity increased by 1.2 NPRS, disability increased by 3.2 RMDQ-24, and HRQoL decreased by 8 RAND-36 points. These associations remained significant and arbitrated by ≤3% in the full adjustment model, and hierarchical models revealed that adding kinesiophobia accounted for an additional 11–12% of variance (ΔR^2^) in each outcome, evidence that fear of movement contributes information not captured by demographic or clinical factors.

The results found that every 10-point increase on the TSK was associated with increases by 1.2 NPRS points (higher pain intensity). This is consistent with a study by Comachio et al. [10], which demonstrated a significant correlation between the TSK and pain intensity (r = 0.18). Where broader psychosocial variables are modeled, the results differ: Corrêa et al. (2022) [26] found that higher odds of kinesiophobia were associated with severe pain, whereas Doménech-Fernández et al. (2025) [12] showed only small correlations once catastrophizing was included. Collectively, these results suggest that kinesiophobia was associated with additional variance in pain intensity beyond the included covariates. These trends suggest that kinesiophobia could add a unique variance to pain intensity, although some of this variance could be shared with catastrophizing.

We found that every 10-point increase in TSK score was associated with a 3.2-point higher RMDQ score (greater disability). This finding aligns with Comachio et al. (2018) [10] study that reported a significant correlation between kinesiophobia and disability (r = 0.39). Further, several other studies have found that while kinesiophobia is the strongest correlate of disability, another cognitive factor, catastrophizing, can actually equal or surpass kinesiophobia when both are modeled. Using a large sample from a tertiary hospital, Doménech-Fernández et al. (2025) [12] found that kinesiophobia, catastrophizing, and sex jointly explained 35% of disability variance, with catastrophizing showing the strongest single correlation. Other studies echo this pattern; higher fear and catastrophizing are repeatedly linked to greater pain and disability [27]. However, our study did not control for catastrophizing; thus, it should be noted that some of the variance explained by the TSK may be shared by catastrophizing. Future research should perform a hierarchical model that enters the TSK and catastrophizes in separate steps to quantify each construct’s unique contribution to disability.

For HRQoL, the results demonstrated an 8-point decrement in the RAND-36 score per 10 points on Tampa, which persisted even after full adjustment. Although HRQoL results may vary across instruments and samples, Comachio et al. (2018) [10] also showed decreases in the SF-36 domains with higher fear. Altug et al. (2016) [28] also found a negative correlation between kinesiophobia and SF-36 subscale scores, which is consistent with a broader impact on perceived health and participation. Importantly, our analysis used a consistent covariate control for each outcome, enabling a more comprehensive understanding of the broader effects of kinesiophobia beyond pain and function. This supports the growing recognition of kinesiophobia as not only a behavioral barrier to movement but also as a psychosocial determinant of overall health status.

Our results correspond with the fear-avoidance model, which posits that people who misinterpret pain as a threat are likely to avoid movement, resulting in disuse, disability, and emotional distress [29]. A previous study reported that kinesiophobia was independently associated with greater disability, frequently more than pain itself, underscoring its central role in the fear-avoidance cycle (Comachio et al., 2018) [10]. However, it is important to differentiate subjective reports of disability from objective evaluations of physical function. Although kinesiophobia is consistently correlated with self-reported disability, its association with performance-based assessments is less robust. For instance, Demoulin et al. (2013) [30] found no significant correlations between TSK scores and objective measurements, such as maximal isometric back extensor strength, endurance as assessed by the Sorensen test, or flexibility assessed by finger-to-floor distance. This dissociation implies that fear of movement may not reflect physical incapacity but rather an internalized perception or feeling of vulnerability or harm, which can limit activity even when physical function is relatively preserved. In this sense, kinesiophobia may act as a psychological barrier that influences behavior independent of biomechanical restrictions. Therefore, clinicians should evaluate functional limitations within a broader biopsychosocial framework, acknowledging that perceived disability may be driven by fear-avoidant expectations and beliefs as much as physical deficiencies.

Importantly, the present study adds to the existing literature by investigating the association of kinesiophobia with pain intensity, functional disability, and HRQoL within the same analytical framework and after adjustment for key demographic and clinical factors. Whereas prior studies have often focused on unadjusted associations or broader psychosocial influences, our findings indicated that kinesiophobia accounted for additional variance in these outcomes beyond the included covariates. From a clinical point of view, these findings suggest that screening for kinesiophobia may be useful as part of routine assessment in people with CLBP.

To the best of our knowledge, this is the first study conducted in Saudi Arabia to investigate the association between kinesiophobia and key clinical outcomes, including pain intensity, disability, and HRQoL, in individuals with CLBP. This study has various strengths, such as the use of validated Arabic instruments, the evaluation of multiple clinically relevant outcomes, and the application of adjusted and hierarchical regression analyses to estimate the independent contribution of kinesiophobia beyond relevant covariates.

However, this study has some limitations. First, some probably relevant factors were not examined in the current study, such as pain duration, physical activity level, in addition to psychological constructs such as pain catastrophizing, psychological distress, and self-efficacy. These factors may impact kinesiophobia, pain intensity, disability, and HRQoL in individuals with CLBP and should be taken into consideration in future research [22,24,25,31]. Second, we did not gather data on the participants’ exposure to therapies such as pain medications or physiotherapy, which could have affected the severity of symptoms or fear-related perceptions. Third, the results should be interpreted with caution regarding their generalizability. Participants were recruited using a non-random convenience sampling approach from a specific clinical setting; hence, the study sample may not be representative of all people with CLBP. Fourth, any conclusions regarding the directionality or causality of the associations were precluded by the cross-sectional design. To better understand the causal association between kinesiophobia and clinical outcomes, future studies should implement prospective designs that account for psychological and treatment-related variables.

## 5. Conclusions

In this cross-sectional study of adults with CLBP, kinesiophobia was associated with higher pain intensity, greater disability, and poorer HRQoL. These associations remained robust even after controlling for demographic and clinical covariates, with kinesiophobia contributing meaningful incremental explanatory value for all outcomes. Although the cross-sectional design hinders causal interpretation, the results highlight the clinical relevance of fear of movement as a nonredundant correlate of symptom burden and functional restriction. Routine evaluation of kinesiophobia may assist in identifying individuals who are more likely to experience poor outcomes and in providing targeted interventions. Future interventions and longitudinal research are recommended to clarify the causal pathways and to explore whether addressing kinesiophobia can improve pain, function, and HRQoL in this population.

## Figures and Tables

**Table 1:** Participants characteristics (*n* = 298).

Variable	*n* (%) or Mean ± SD
Age (years)	38.7 ± 13.2
Sex	
Female	173 (58.0%)
Male	125 (42.0%)
Body mass index	
Underweight (<18.5 kg m^−2^)	17 (5.7%)
Normal (18.5–<25)	99 (33.2%)
Overweight (25–<30)	86 (28.9%)
Obese (≥30)	96 (32.2%)
Marital status	
Single/Divorced	133 (44.6%)
Married	165 (55.4%)
Education level	
High-school or less	67 (22.5%)
College (Bachelor)	201 (67.4%)
Post-graduate	30 (10%)
Employment status	
Employed	145 (48.7%)
Unemployed/Retired	153 (51.3%)
Current smoker	
Yes	54 (18.1%)
No	244 (81.9%)
Any chronic disease	
Yes	86 (28.9%)
No	212 (71.1%)
Tampa kinesiophobia (0–68)	44.5 ± 5.4
Pain intensity (NPRS 0–10)	6.5 ± 2.1
Functional disability (RMDQ-24)	7.7 ± 5.7
HRQoL (RAND-36 composite)	47.8 ± 12.9

SD: Standard Deviation.

**Table 2:** Unstandardized regression coefficients (per 10-point increase in Tampa score).

	Model 1 ^a^			Model 2 ^b^		
Outcome (DV)	β (95% CI)	*p*	Adj R^2^	β (95% CI)	*p*	Adj R^2^
Pain intensity (NPRS-10)	1.19 (0.61–1.77)	<0.001	0.17	1.17 (0.55–1.79)	<0.001	0.18
Disability (RMDQ-24)	3.27 (2.14–4.41)	<0.001	0.15	3.24 (2.05–4.43)	<0.001	0.14
HRQoL (RAND-36)	−7.99 (−11.0–−4.98)	<0.001	0.17	−7.98 (−11.1–−4.81)	<0.001	0.17

^a^ Model 1 controls for age and sex. ^b^ Model 2 additionally controls for BMI, marital status, education, employment, smoking, and chronic disease. Abbreviations: β, unstandardized regression coefficient; CI, confidence interval; Adj R^2^, adjusted coefficient of determination; HRQoL, health-related quality of life; NPRS, Numeric Pain Rating Scale; RMDQ, Roland-Morris Disability Questionnaire.

**Table 3:** Hierarchical ΔR^2^ when kinesiophobia is entered after all covariates (step 2).

Outcome	Step 1 ^a^	Step 2 ^b^	ΔR^2 c^	F-Change (1, 287)	*p*
Pain intensity (NPRS-10)	0.07	0.18	0.11	36.5	<0.001
Functional disability (RMDQ-24)	0.02	0.14	0.12	43.9	<0.001
HRQoL (RAND-36)	0.06	0.17	0.11	38.2	<0.001

^a^ Step 1 = age, sex, BMI, marital status, education, employment, smoking, chronic disease. ^b^ Step 2 = all covariates entered in step 1 + Tampa. ^c^ Increment from Tampa. Abbreviations: β, unstandardized regression coefficient; CI, confidence interval; ΔR^2^, change in coefficient of determination; HRQoL, health-related quality of life; NPRS, Numeric Pain Rating Scale; RMDQ, Roland-Morris Disability Questionnaire.

## Data Availability

The dataset supporting the findings of this study has been deposited in Zenodo and is publicly accessible at https://doi.org/10.5281/zenodo.19466280.
